# Is Spontaneous Preterm Prelabor of Membrane Rupture Irreversible? A Review of Potentially Curative Approaches

**DOI:** 10.3390/biomedicines11071900

**Published:** 2023-07-04

**Authors:** Bianca Mihaela Danciu, Marina Ruxandra Oţelea, Marian Augustin Marincaş, Maria Niţescu, Anca Angela Simionescu

**Affiliations:** 1 Carol Davila University of Medicine and Pharmacy, 050474 Bucharest, Romania; bianca-mihaela.danciu@drd.umfcd.ro; 2 Department of Obstetrics, Gynecology and Neonatology, “Dr. Alfred Rusescu” National Institute for Maternal and Child Health, 127715 Bucharest, Romania; 3 Clinical Department 5, Carol Davila University of Medicine and Pharmacy, 050474 Bucharest, Romania; marina.otelea@umfcd.ro; 4 First Department of Surgery, Bucharest Oncological Institute Prof. Dr. Alexandru Trestioreanu, Carol Davila University of Medicine and Pharmacy, 022328 Bucharest, Romania; marian.marincas@umfcd.ro; 5 Preclinical Department 3, Complementary Sciences, Carol Davila University of Medicine and Pharmacy, 020125 Bucharest, Romania; maria.nitescu@umfcd.ro; 6 Department of Obstetrics and Gynecology, Filantropia Clinical Hospital, Carol Davila University of Medicine and Pharmacy, 050474 Bucharest, Romania

**Keywords:** fetal membranes, amnios, premature rupture of membranes, healing membrane, amniopatch

## Abstract

There is still no curative treatment for the spontaneous preterm prelabor rupture of membranes (sPPROM), the main cause of premature birth. Here, we summarize the most recent methods and materials used for sealing membranes after sPPROM. A literature search was conducted between 2013 and 2023 on reported newborns after membranes were sealed or on animal or tissue culture models. Fourteen studies describing the outcomes after using an amniopatch, an immunologic sealant, or a mechanical cervical adapter were included. According to these studies, an increase in the volume of amniotic fluid and the lack of chorioamnionitis demonstrate a favorable neonatal outcome, with a lower incidence of respiratory distress syndrome and early neonatal sepsis, even if sealing is not complete and stable. In vivo and in vitro models demonstrated that amniotic stem cells, in combination with amniocytes, can spontaneously repair small defects; because of the heterogenicity of the data, it is too early to draw a thoughtful conclusion. Future therapies should focus on materials and methods for sealing fetal membranes that are biocompatible, absorbable, available, easy to apply, and easily adherent to the fetal membrane.

## 1. Introduction

Nowadays, spontaneous preterm prelabor rupture of membranes (sPPROM), occurring during 24–37 weeks of gestation before the onset of contractions, and spontaneous labor are potentially viable, while sPPROM before 24 weeks is generally considered previable [1]. Midtrimester sPPROM incidence is estimated to occur in <1% of pregnancies [2,3], with survival rates ranging from 51.7% to 100%, depending on gestational age [3]. There are several possible approaches to sPPROM: expectative management, labor induction, or termination of pregnancy before reaching viability [4]. Previable sPPROM is associated with maternal morbidities and frequently requires blood transfusion or admission to intensive unit care [5]. This also leads to infant complications such as stillbirth, death in the delivery room, cerebral palsy, or admission to neonatology intensive unit care [3]. Several risk factors for previable sPPROM, such as high-risk pregnancies, e.g., multiple pregnancies and advanced (>35 years old) or older maternal age (>40 years old), are described as comorbidities [5,6]. Depending on latency, the gestational age at birth, and associated conditions, sPPROM between 24 and 37 weeks results in high rates of neonatal mortality and morbidity, prematurity, sepsis, or cord prolapse [7]. Placental abruption and chorioamnionitis, other frequent complications, endanger the mother’s life [8]. After sPPROM, the median latency to spontaneous labor is one week, but it tends to shorten in more advanced pregnancies [9,10].

Fetal membranes (the amniotic and the chorionic membranes) begin to develop on the 6th day of gestation. They expand during the entire pregnancy to protect the fetus throughout the pregnancy. In the first trimester, they include the chorion, which is a thicker membrane in close contact with the endometrial tissue by decidua and the placenta, and the amnion, which is thinner and in contact with the amniotic fluid. Before 15 weeks, the amnion is separated by an intermediate layer, a collagen-rich extracellular matrix (ECM) that contains amnion mesenchymal stromal cells in the decidual chorion; after 15 weeks, the amnion and chorion are healed. The amniotic membrane is poorly vascularized and has three layers: cuboidal and columnar epithelium layers, basement membranes, and stroma with collagen and fibroblast cells. The decidua is extraordinarily rich in maternal blood vessels and immune cells. The basement layer of the amniotic membrane is one of the thickest human membranes and protects the fetus during pregnancy [11]. Neither of them is innervated. The human amniotic membrane produces anti-inflammatory cytokines and upregulates genes associated with wound healing after surgery [12]. It is also an important source of human amniotic mesenchymal stem cells, which have an intrinsic ability for tissue regeneration and anti-inflammatory properties [12,13].

The main causes of sPPROM are represented by amniotic fluid infection, especially chronic bacterial colonization [14], mechanical amniotic stretch, increased oxidative stress, and inflammation of the amniochorial membranes [15]. Studies have reported the presence of commensal microbes, bacterial, fungal, and viral DNA in the human placenta [14], umbilical cord [16], meconium [17], and amniotic fluid [18] after the second trimester of pregnancy as physiologic fetal microbiota that contribute to perinatal human gut colonization. 

Intra-amniotic inflammation causing sPPROM may result from the microbial invasion of the extra-amniotic compartments and can be triggered by both infectious and noninfectious causes (trauma, ischemia, and abruptio placenta) [19]. Inflammation and inflammatory triggers at term initiating physiologic labor are very different from inflammatory signals before 34 weeks, which increase oxidative stress and accelerate premature cellular senescence, senescence-associated inflammation, and proteolysis, predisposing the amniotic membranes to premature rupture [20]. Another possible explanation of the pathophysiological process that takes place in these cases is the “strain hardening” described by Lavery [21], which causes the loss of elasticity of the membranes and increases their friability [22]. Increased thrombin activity in plasma and amniotic fluid [23] and decreased collagen levels in amniotic membranes are frequently associated with PPROM. The level of relaxin was significantly high in all cases of preterm PPROM with sterile amniotic fluid. Relaxin increases local levels of metalloproteinases and tissue plasminogen activators that lead to extracellular matrix degradation and rupture [22]. Membrane weakening and spontaneous PPROM are consecutive to the amniotic fluid inflammation. This can result after exacerbated production of hormones, cytokines (interleukins IL-1β, IL-6, 8, 18, and TNFα), and proteolytic enzymes in patients with chorioamnionitis and PPROM in the uterus, amniotic fluid, membranes, and placenta [24]. TNFα and IL-1β increase matrixmetalloprotease-9 (MMP-9) and PGE2 production in human chorioamnion and amnion epithelial cells, which is associated with fetal membrane rupture [25].

A thickened membrane measured by high-frequency ultrasound probes, which is probably due to inflammatory processes, may increase the risk of premature rupture of membranes, while a thickness ≤1.2 mm is a negative predictive factor for premature membrane rupture [26].

There is evidence that amniotic membrane transplantation is an alternative to the regeneration or healing of multiple tissues in adults, such as the cornea, skin from burns, or diabetic foot ulcers, because of biological properties (anti-inflammatory, antineoplastic, lack of immunogenicity, and vascularization) that favorize recovery [27,28,29,30].

The ability of amnio-chorionic membranes to heal or to regenerate spontaneously or after sealing appears to be a better physiologic solution than conservative management after PPROM and avoids negative maternal and fetal outcomes due to infection and prematurity. 

Iatrogenic mechanical PPROM performed by minimally invasive fetal surgery for severe complications occurs in 27.4% of cases after fetoscopic laser ablation for twin-to-twin transfusion syndrome [31], in 28% of cases of umbilical cord ligation and tracheal occlusion [32], and in 50% of cord occlusion in monochorionic diamniotic twins [33]. Fetoscopic surgery, intrauterine fetal shunting for lower urinary tract obstructions (LUTO), and pleural effusions are associated with mechanical PPROM in variable proportions, between 0 and 100%, depending on the complexity of the procedure. While minimally invasive procedures such as amniocentesis or fetoscopic surgery procedures increase the rate of PPROM, they have become more frequent due to the increase in maternal age, improved technology, and procedure outcomes. Most invasive procedures are performed in the second trimester of pregnancy, while sPPROM frequently occurs at an early gestational age. The perinatal outcome is generally improved after surgery [34]. Although the cause of PPROM in the early second trimester differs between spontaneous and iatrogenic cases, its consequences may be comparable. The gestational age at delivery, the management of PPROM, together with the duration of oligohydramnios that increase the risk of lung hypoplasia, chorioamnionitis, and fetal exposure to infection and inflammation, have significant implications for perinatal mortality and early childhood complications [35]. The defect in sPPROM is hard to delineate, whereas the defect in iatrogenic PPROM depends on the size of the instrument, and it is clearly delineated. After fetal surgery, chorioamniotic membranes separate in up to 30% of patients without spontaneous healing, commonly leading to iatrogenic PPROM [36]. The defect in sPPROM is located over the cervical ostium and rarely heals by itself. By contrast, the defect in iatrogenic PPROM occurs where the device is inserted. After amniocentesis or chorionic villus sample without infection, spontaneous healing often occurs. After fetal surgery, spontaneous healing is far less frequent. Studies on iatrogenic PPROM have focused on insertion techniques to induce membrane sealing during fetoscopic surgery at the time of the primary intervention [37,38].

Before 24 weeks of gestation, PPROM managed expectantly has a survival rate ranging from 26 to 80%, and almost one-third of babies have developmental delays [39] and long-term disabilities, including chronic respiratory diseases, cardiovascular diseases, behavioral effects, or sequela [40]. After 28 weeks of gestation, PPROM has a survival rate of almost 100% due to advances in neonatology care [41].

Two legitimate questions arise: (1) are the available sealing techniques able to heal and maintain the membrane recovery, stopping the pathogenic mechanism that was involved in or reestablishing the amnion functions? and (2) why do amniotic membranes taken at birth contribute to the sealing of different tissue and not to the automatic recovery process of the amniotic membrane itself? To address these questions, we provide an overview of the current knowledge on the healing mechanism and outcomes after healing and sealing of spontaneous PPROM, including technical means used and pregnancy outcomes. 

## 2. Materials and Methods

A literature search based on articles published between 2013 and 2023 on the survival of newborns after spontaneous and iatrogenic PPROM were sealed, as well as on animal or tissue culture models of sealing membranes after PPROM using different materials, was conducted using MEDLINE, WEB OF SCIENCE, and SCOPUS databases using the PubMed and OVID search engines in English, without restriction on the type of publication. A manual search of relevant articles was conducted by two of the authors (B.M.D. and A.A.S.). We also considered animal studies for their possible contribution to the scientific knowledge needed for the development of the treatment. Free text searches were performed with combinations of the following keywords from the title or abstract: “sealing amniotic membranes” OR “amniopatch” OR “healing amniotic membranes” AND “fetal’’. Additionally, reference sections of eligible studies were hand-reviewed to identify potentially eligible studies. Relevant studies were identified for each question by reviewing titles and abstracts. All references were independently screened by three authors (A.A.S., B.M.D. and M.R.O.). Disagreement on the eligibility of a study was resolved by discussion until a consensus was reached. A reference list was built after an agreement among the authors, and full papers were also used to identify additional papers for review. If abstracts referred to one or more cases about the healing or sealing of amniotic membranes in pregnancy, full-text papers were reviewed. As all data were available from medical literature databases and did not involve retrieving any sensitive data, the study was exempt from the Institutional Ethics Committee review. 

Inclusion Criteria: Our search included articles published in the last 10 years that reported on live newborns delivered after spontaneous premature rupture of membranes that were sealed. Randomized trials, descriptive and comparative studies on human data, and prospective and retrospective case reports or case series on spontaneous PPROM sealed followed by live babies born were considered eligible for inclusion. We focused on clinical cases with obstetrical outcomes. Secondly, we included papers related to the description of animal or in vitro human membrane models focused on healing or sealing membranes and the closure of the defect during pregnancy using different biological or synthetic materials, accompanied by fluid leakage evaluation and/or fetal outcomes. 

Exclusion Criteria: We excluded articles concerning iatrogenic PPROM in humans, including post-fetal surgery or invasive maneuver, and studies with overlapping data, opinions, letters, conference abstracts, and review articles without clinical cases. Additionally, we excluded studies on different models evaluating different characteristics of the sealing product without mimicking the pregnancy conditions, for example, insertion and adhesion instruments, the technique of insertion, and physical parameters of sealing material evaluation, e.g., time to seal, pressure applied, adhesion strengthened, or open fetal surgery cases. 

This systematic literature review was performed according to the Preferred Reporting Items for Systematic Reviews and Meta-Analyses (PRISMA) guidelines [42] where applicable. Grey literature, unpublished literature, and electronic ahead-of-print articles were not included.

We recorded reported information concerning the subjects, gestational age at spontaneous rupture, the technique used, the sealing material, and the final obstetrical and neonatal outcomes. Obstetrical outcomes include gestational age at birth, term prolongation after membrane rupture, and maternal complications. Neonatal and perinatal outcomes include live or stillbirth and short and long-term complications. All included cases concerned sPPROM without clinical chorioamnionitis or inflammatory signs, without vaginal bleeding, and without fetal abnormalities. Clinical chorioamnionitis was defined as maternal fever of 37.8 °C or more, plus one or more of the following signs: uterine tenderness, malodorous vaginal discharge, maternal serum white blood cell count of more than 15,000 cells/mm^3^, maternal tachycardia (>100 beats/min), and fetal tachycardia (>160 beats/min).

### Assessment of Reporting Quality

The quality of the included papers on human cases of PPROM and sealing or healing methods was assessed using the Downs and Black checklist and score ranges based on 27 questions [43]. Studies with scores above 20 were classified as high quality, 15–19 as moderate, and below 14 as low quality (Supplementary Materials). The quality of the included studies on animal models was assessed using the scale reported by Kringe et al. [44] based on four categories and 24 questions: subjects’ details, study details and design, internal studies validity, and quality of outcome analysis, containing 6 items and 24 items. Each study was given a score from 0 (lowest quality) to 24 (highest quality), with each category having a quality value of 0 (lowest quality) to 6 (highest quality). Studies with a score above 16 were classified as high quality, 11–15 as moderate, and below 10 as low quality. Three reviewers (A.A.S., M.R.O. and M.N.) independently evaluated the quality of each study. 

Data were presented in a descriptive way, including the reported cases of perinatal outcomes: live birth, mortality, associated morbidities, and the evaluation efficiency and safety of the patch. Because of the empirical and non-reproducible nature of the cases, statistical analysis and comparisons were not considered. 

## 3. Results

**Figure 1:**
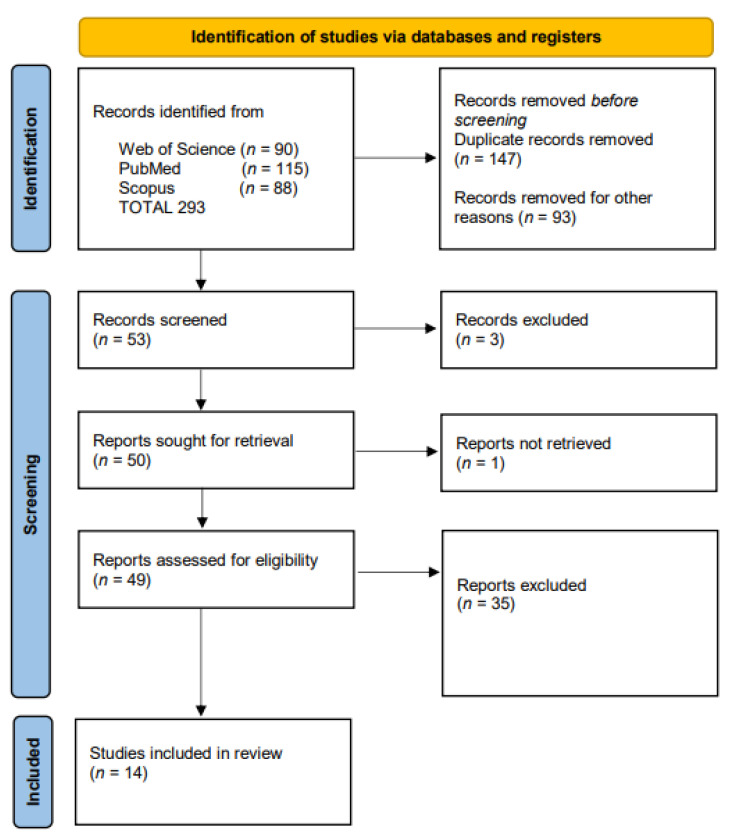
Flow diagram showing the process of searching the databases and publication screening.

The electronic search generated 90 articles from the Web of Science core collection, 115 articles from the MEDLINE-PubMed database, and 88 articles from the Scopus database. After reading the titles and abstracts, 53 articles were selected for further investigation. The abstract and full text of 52 publications were read; one full-text article was not found. Thirty-eight articles were excluded as follows: 1 article described the same cases, 1 article was considered inappropriate research practice and raised concerns [45], and 36 articles did not meet the inclusion criteria (Figure 1). One previous systematic review on this topic was excluded [46] because the authors described the same cases as this review and included one article with iatrogenic PPROM and one article that raised concerns. Finally, 14 studies met the eligibility criteria and were included and analyzed in this systematic review [47,48,49,50,51,52,53,54,55,56,57,58,59,60].

Four studies [47,48,49,50] on the spontaneous premature rupture of membranes analyzed the outcomes for 141 pregnancies and sealing and healing methods: 82 cases using an amniopatch with autologous or donor platelet concentrates and cryoprecipitate inserted by the ultrasound-guided amnioinfusion technique and 59 cases using an immunologic sealant or a mechanical cervical adapter for sealing (Table 1). 

An amniopatch was used after serial amnioinfusion in case of continuous amniotic fluid leakage and anamnios/oligohydramnios [47,49,50]. In all studies, patients with regular uterine contractions or vaginal bleeding, signs or symptoms of clinical chorioamnionitis, or major fetal congenital anomalies were excluded. In all cases, bacteriology for cervical infection was performed before the application of the sealing method. Sealing was used in cases with or without cervical insufficiency. Table 1 summarizes included studies on amniotic membrane sealing involving human models. 

An increase in the volume of amniotic fluid after the sealing procedure demonstrated a favorable neonatal outcome compared to the conservative management group in terms of lower incidence of respiratory distress syndrome and early neonatal sepsis, even if sealing was not complete and stable. From their case–control retrospective series on previable sPPROM, Kwak et al. reported the complete sealing of membranes with restoration of amniotic fluid in two cases (from 8 procedures) and seven cases of live births (75%). One patient with spontaneous PPROM at 17 2/7 weeks of gestation and amniopatch performed after 5 weeks delivered at term, but in a second case, with cervical insufficiency, the new rupture of membranes after complete membrane sealing was reported. Six babies (from seven cases) were discharged. In the amniopatch group, the authors found a lower incidence of respiratory distress syndrome and a lower incidence of neonatal sepsis when compared with the conservative management group [47]. 

A 2016 Cochrane review of randomized trials of membrane sealing studies [48], analyzing two studies from 1994 [61] and 2011 [62], concluded that there are insufficient clinical data to evaluate sealing procedures and performance for PPROM, including spontaneous PPROM. Dam et al. [62] addressed the etiologic mechanism of sPPROM using a proapoptotic bax gene P-53 inhibitor, myristoleate with antimicrobial peptides, α plus β defensins, neutrophil defensins, and cytokine IL-10. Vaitkiene et al. used a mechanical device to close the cervix and prevent amniotic fluid leakage without an increase in maternal or fetal complications [61]. There was no clear difference between the mechanical sealing group and the standard management cases in relation to the incidence of neonatal sepsis or chorioamnionitis. Compared to standard care, the oral immunological membrane sealant reduced preterm birth at less than 37 weeks and neonatal death, but there was no difference in neonatal sepsis [48].

In four cases of the Sung et al. series, risk factors from PPROM were twin pregnancy, cervical insufficiency, and uterine malformation. No serious maternal (hematomas, chorioamnionitis, and placenta abruption) or fetal complications (fetal death and stillbirth) were associated with amniopatches. There was more than one procedure for patients with a second rupture of membranes. A maximum vertical pocket of amniotic fluid of 1.8 cm was associated with the success rate of sealing. Two babies were born at term, and in 11.8% (2/17) cases, no fluid leakage was reported. The neonatal outcome of the live-born infants between the amniopatch group and the conservative management group were comparable. The period of the prolongation of pregnancy was 30 days (3–123) in the group of sealing membranes when compared with 14 days (0–67) in the conservative management group. In the series by Sung et al., the occurrence of clinical chorioamnionitis was associated with the failure of sealing membranes [49]. 

No complete closure of the amniotic defect was obtained by Ferianec et al., but in most patients, they achieved an increase in AF volume production with a different duration of the interval without AF leakage. From 53 cases of amniopatch use, complete closure of the amniotic defect was observed in 6 women (12%), 35 babies were live-born (66%), and 33 babies survived (62%) and were discharged [50].

Our review analysis included 10 preclinical studies in animal models of pregnancy rupture of membranes (rabbit, mouse, micropig, rats, and ewe) (Table 2) and ex vivo and in vitro studies on the human fetal membrane rupture mechanism mimicking a pregnancy, filled with amniotic fluid (Table 3).

Table 3 presents the selected studies on in vitro fetal amniotic membrane sealing models.

Amniotic epithelial stem cells derived from the amnion and extracellular mesenchymal matrix are reported as a curative mechanism to heal the amniotic membrane after <1 mm of spontaneous rupture [54,57].

Various techniques and materials have been tested for fetal membrane plugging and bonding: mussel glue alone or combined with decellularized amnion membranes [51], an amniopatch, different tissue adhesives and sealants [57,58], and Arg-1 positive M2-macrophages [52]. MG performance in sealing fetal membranes in the rabbit model was comparable to that of FG. MG has better mechanical and adhesive proprieties, is less susceptible to proteolytic degradation, and has no inflammation inside the glue [51]. After applying Arg-1 positive M2-macrophages in the small rupture, the average complete closure of amnion was 83% at 24 h and 98% at 72 h. However, in the large rupture, the closure of amnion was 7% at 24 h and 48% at 72 h. The closure of choriodecidua was impaired significantly relative to amnion with 61% and 78% at 24 and 72 h, respectively, in the small rupture model. In the large rupture, the choriodecidua did not heal at 24 h and only 16% at 72 h. IL-1β and TNF induce epithelial–mesenchymal transition, which enhances tissue repair and cellular migration for small ruptures, but after a large rupture, there is no mesenchymal cell migration and matrix deposition. Embryonic wound healing is rapid and complete compared with skin healing or other adult tissues [52]. An animal model for the application of AMED showed that AMED could mimic the main extracellular matrix, has better biocompatibility than DAM, and it is implicated in the functional restoration of the amniotic membrane that prevents fluid leakage. AMED is correlated with better fetal lung development. The amniotic fluid presence rate was 56% for the AMED group in comparison with 49.80% for the amniopatch group [53]. Condensed collagen from human amniotic membranes tested by Engels has good biocompatibility but is difficult to manipulate [54]. The elastic mussel glue tested by Avilla-Royo was elastic and resistant to spontaneous or proteolytic degradation, determining mild immune cell inflammation after 10 days. This glue firmly adhered to both the myometrium and the fetal membranes, and no cellular infiltration was observed [56].

Lee et al. recently reported that human amniotic membranes and amniotic epithelial stem cells have pluripotent proprieties, including regeneration and promoting cells and natural membrane healing [57]. In 2018, Lee et al. tested an amnion-analogous medical device containing amniotic membrane gel and found that the device is easy to apply and heals wounds two times faster than an amniopatch [53]. Kondoh et al. tested a gynecologic cervical sealant used as an intracervical elastomeric sealant, Hydrophit [58]. No effect on fetal survival after sealing membranes with Lyostypt, Lyostypt soaked in fibrinogen concentrate, condensed collagen, Tissuepatch, and Duraseal was reported, but low fluid leakage was reported after membrane sealing with Tissuepatch in the rabbit model [54]. Many sealants have rapid degradation and poor tissue adhesion. This is why researchers are looking at materials with strong amniotic adherence that are able to withstand pressure, are biocompatible, and can maintain mechanical elasticity. For example, peptide amphiphiles (PAK3) conjugated with ligands, already used for cell-adhesion or regeneration, form a structural interface after contact with amniotic fluid, which grows and plugs the defect with a multi-layered resistant structure, providing stability after 4 days [59]. An ultrafast photoresponsive hydrogel tested by Zhao et al. showed robust tissue adhesion, biocompatibility and rapid tissular integration, mechanical proprieties, typical shear-induced gel-fluid transition behavior, and rapid self-healing with reepithelialization. This sealant demonstrated a leakage profile similar to intact fetal membranes [55].

The addition of a shape memory for the plugs also ameliorates expansion proprieties and may determine a better adherence to the membrane defect. The crosslinked collagen plugs seal the defect after 1 h and maintain closure.

As the main differences from humans, animal models are represented by shorter gestation time and vascularized membranes [60]. Sealing materials applied in animal models have demonstrated a pathway of activation of the innate immune system and an increase in amniotic macrophages, which release limited and well-localized cytokines and growth factors such as TNF and IL-1β [52].

The quality of the studies was high for in vitro models and moderate to low for human studies with incomplete outcomes, no statistical power, and little description of detailed data (Supplementary Materials). No representative samples were tested; sealing methods were used most in animal models or in vitro tests. 

## 4. Discussion

The purpose of this article was to review clinical cases of sPPROM and curative approaches to prolong pregnancy that have the potential for adoption in clinical practice. We focused on the curative approaches of treatment for spontaneous premature prelabor rupture of membranes. A total of 141 pregnancies and sPPROM used sealing and healing methods: 82 cases used an amniopatch with autologous or donor platelet concentrates and cryoprecipitate inserted by ultrasound-guided amnioinfusion technique, and 59 cases used an immunologic sealant or a mechanical cervical adapter. Large amniotic fluid volume and the absence of the occurrence of clinical chorioamnionitis were associated with the healing success group. The neonatal outcome was more favorable in the sealing groups compared to the conservative expectant management group in terms of respiratory distress syndrome and chorioamnionitis; amniotic fluid restauration, complete or partial closure of membrane defect, and prolongation of pregnancy for more than 24 h were obtained. Spontaneous healing of small defects associated with cell regeneration and natural healing membranes was attributed to amniotic epithelial stem cells [57]. Amniotic stem cells and amniocytes, in combination with fibrin glue, peptide amphiphiles, and elastomeric sealants, enhance adhesion, cell migration, and regeneration and can repair FM defects after trauma.

Identifying the appropriate treatment for PPROM requires good knowledge of the mechanisms that lead to amniochorionic membrane rupture, in labor or prematurely, whether they are genetic in nature (apoptosis of the amniotic epithelium and chorionic trophoblast), epigenetic (changes in the structure of MMP and TIMP promoter genes), physical (excessive stretching membranes), inflammatory (cytokines released by immune cells lead to high levels of oxidative stress), or hormonal (increased levels of relaxin).

While human fetal membranes are almost avascular, thus incapable of initiating hemostasis, platelet activation, fibrin deposition, or inflammation, and therefore wound healing, spontaneous “resealing” defined by the cessation of leakage of amniotic fluid has been documented in 3–8% of cases of small rupture size, with the possible restoration of amniotic fluid volume [57,58,59,60,61,62,63]. The entire sealing and healing process depends on gestational age. Amniocytes obtained from digested fresh human fetal membranes, which are cells obtained at earlier gestational ages, show higher proliferation rates and faster closure of the central defect [52,64]. It was recently reported that the difference between term and preterm tissue for the expression of genes associated with the inflammatory response is only present for mesenchymal cells for the amnion [65]. The mechanism of PPROM is sometimes self-limited, involving retraction, sliding, contraction, and scarring in the myometrium, but no cell proliferation of the fetal membranes without an external stimulus was noted [52,66]. Using a mouse model, Mogami H et al. demonstrated that the small rupture of membranes (0.47–0.91 mm) induced transient upregulation of cytokines and significantly decreased the membrane defect and healing process after 24–72 h. Large membrane ruptures (more than 0.91 mm) determine partial healing by partial healing of choriodecidua and by an increase in proinflammatory cytokines in the amniotic membrane. Fetal macrophages originating from the amniotic fluid are recruited to the wounded ruptured amnion, where macrophage adhesion molecules are highly expressed. Recruited macrophages release limited and well-localized amounts of IL-1β and TNF, which facilitate epithelial–mesenchymal transition (EMT) and epithelial cell migration. Arg1+ macrophages dominated within 24 h. However, migration and healing of the amnion mesenchymal compartment remained compromised. Collagen type 1 injected at the ruptured site between the myometrium and fetal membranes resulted in a collagen layer with plenty of macrophages and determined almost complete healing of the sterile ruptured amniotic membranes. A 40% rate of healing of the chorionic layer and consecutively of the amniotic layer after phosphate-buffered saline injection at the site of rupture were reported [66,67]. Galectins, which are carbohydrate-binding proteins in the extracellular matrix, and crucial for implantation and pregnancy maintenance, may also play a role in the remodeling phase of healing amniotic membranes. Galectins act in the immune response at the amniotic interface, stimulate monocytes to enhance the expression levels of Akt, PI3K, and PPAR-γ, and turn on the M2 polarization of macrophages. Galectins interact with epithelial–mesenchymal transition, which induces a healing mechanism [68].

PPROM before 34 weeks represents a great challenge for the obstetrician, neonatologist, and pediatrician and has a very high healthcare cost. Given the large number and diversity of pathophysiological processes involved in the rupture of membranes, several treatment methods have already been tested. Tocolysis, antibiotic therapy, corticosteroids, and magnesium sulfate are just a few. Other “curative” treatment options include defect closure techniques such as amniopatches, amnion cell engineering, collagen plugs, fibrin sealants, mussel-mimetic tissue adhesives, immunological supplements, polymeric film, mechanical cervical adapters, gelatin sponges, and lasers. We found in the above-mentioned studies that, in general, the strict application of resealing methods is not effective. The association of resealing with amnioinfusions, corticotherapy, and/or tocolysis has more beneficial effects on neonatal evolution. Considering the fetal outcome, extending the period during which the fetus develops in utero decreases the rate of occurrence of fetal complications.

Amniotic membranes are avascular, thus incapable of initiating hemostasis, platelet activation, fibrin deposition, or inflammation. Although they represent only 20% of the depth of fetal chorioamniotic membrane, they are one of the strongest tear-resistant tissues, with multiple roles in healing and regeneration. Starting from what is known about the healing and adhesion of membranes regarding their adjuvant capacities in the regeneration of other tissues, new models of their adhesion techniques should be developed.

Both in vitro models and those based on clinical cases have demonstrated that an amniopatch (autologous platelet concentrates and cryoprecipitate) inserted by the ultrasound-guided amnioinfusion technique 2–10 days after membrane rupture can seal the membrane defect, especially for small ruptures (<20 mm).

There is no preventive or curative treatment for the premature rupture of membranes. There are widespread treatment protocols for the adaptation of the newborn, but none of them are effective in preventing premature birth after PPROM. If there was an option to avoid their rupture or to help them heal after rupturing, prematurity, probably the most common complication of pregnancy, could be avoided, thus leading to fewer complications for both the mother and the fetus. The problem is that none of the current therapies practically target the cause, and they only succeed in delaying birth without being a real solution to the true pathology.

In 1985, Vintzileos demonstrated that a decrease in amniotic fluid volume after membrane rupture may impair pregnant women’s ability to combat amniotic infections [69]. Normal amniotic fluid volume is important for fetal growth and fetal movement and also protects the umbilical cord and placenta from compression. Several studies have associated oligohydramnios and infection after preterm rupture of the membrane in high-risk pregnancies, especially before 26 weeks, with a significant risk factor for perinatal infection and fetal distress, cesarean delivery, and neonatal death [70,71]. That is why many cases of sealing membranes are accompanied by amnioinfusion.

The main limitation of the study is the varied data and differences concerning indications and selection criteria, gestational period, multifetal pregnancy in animal models, and also in the model of amnion and chorion vascularization and fusion. Animal models using pregnant mice, rabbits, ewes, or sheep have strengthened the causal link between intrauterine infection or high intrauterine pressure and spontaneous rupture of the membrane, but they differ sufficiently from women in both placentation and the hormonal events surrounding parturition. Non-human primate models would be the most applicable animal models for the long-term testing of sealing materials for iPPROM, but we did not find any study on this [51]. Additionally, the in vitro models studied here concern term membranes. It was suggested that preterm membranes are stiffer and harder, and the extracellular matrix differs depending on gestational age [72]. Sealing methods after spontaneous premature rupture of the membrane cannot be studied in a metanalysis or a systematic review because of the heterogenicity of data and the rarity of reported cases, differences in experimental methods, and outcomes; additionally, there is a lack of consensus or guidelines for curative treatment.

The strength of the updated analysis is the systematic review of recent literature to provide a real answer to the difficult problem of finding an effective curative treatment for sPPROM.

## 5. Conclusions

Although intrauterine surgery is promising, this highly depends on the availability of a PPROM repairing method if this condition has already appeared. Until now, none of the existing techniques have shown their superiority, and thus, all have remained in the study stage, with most of them not yet tested in humans. At present, there are no safe and effective treatments for healing PPROM. It is too early to draw a conclusion, and future therapies should focus on new materials for sealing fetal membranes that are non-toxic, rapidly absorbable, available, easily applied to the rupture site, and easily adherent to the fetal membrane. Once treatment strategies have been optimized and safety issues resolved, their efficacy should be evaluated in randomized controlled trials.

## Figures and Tables

**Table 1:** Studies that reported human obstetrical outcomes after membrane sealing technique for sPPROM.

Article (Author *, Year, Country)	Type of Study	Gestational Age at PPROM	Number of Subjects/ Procedures	Treatment and/or Evaluation before Sealing Method	Technique Used and Sealing Material	Treatment and Evaluation after Sealing Method	Outcomes
Kwak, 2013 [47], South Korea	Case–control (amniopatch versus conservative management group)	17–23 weeks	7/8	Antibioprophylaxis for 7 days and Amniotic fluid	Amniopatch with autologous platelet concentrates and cryoprecipitate inserted by ultrasound-guided amnioinfusion technique after amnioinfusion after 7 days	Corticotherapy for pulmonary maturation 24 h after sealing	Lower incidence of respiratory distress syndrome and lower incidence of neonatal sepsis in the amniopatch group.
Crowley, 2016 [48], India, Lituania	Randomized and quasi-randomized control trial	less than 37 weeks	59/16 cases cervical adapter and 43 cases oral immunological membrane sealing	Cervical bacteriology before cervical adapter Amnioseal: antibioprophylaxy, tocolysis and prophylactic corticosteroids	Cervical adapter (mechanical sealing) in the 24 h after PPROM. Oral immunological membrane Amnioseal (TNS Meryl Pharma) (a combination of matrix metalloproteinases inhibitors, cytokines and defensins)- two capsules after 3 h, followed by two capsules 8 hourly for up to 72 h. Maintenance dose was one capsule twice daily for 15 days and one capsule daily for another 15 days	Cervical adapter: Evaluation for AFI and chorioamnionitis Amnioseal: evaluation for chorioamnionitis, AFI and liver function	Cervical adapter is useful for an increase in amniotic fluid volume
Sung, 2018 [49] South Korea	Cohort, retrospective	15–23 weeks	17/21	Antibiotherapy for 2 days Measurement of amniotic fluid volume by MVP	Amniopatch autologous after 2 days of conservative treatment, ultrasound-guided amnioinfusion, using 20–22 gauge needles, of the platelet concentrate followed by cryoprecipitate	Antibiotic therapy Daily measurement of amniotic fluid +/− Corticotherapy or tocolytic	Lower incidence of respiratory distress syndrome and early neonatal sepsis in the amniopatch group
Ferianec, 2022 [50], Slovakia	Descriptive	19 + 3–22 weeks. Cervical length of more than 25 mm	53	NS	Amniopatch platelets and fresh frozen plasma from donors, transamniotic, after minimum 10 days post rupture, amnioinfusion	NS	No maternal/fetal complications directly related to the amniopatch procedure

* The first author’s name was noted. Legend: AFI amniotic fluid volume; MVP: maximum vertical pocket; NS: not specified.

**Table 2:** Studies on animal models using different materials and techniques for sealing and repairing amniotic membranes.

Article (Author *, Year, Country)	Preclinical Model/Type of Study	Length of Gestation	Intervention/Mechanism	Diameter of Defect	Performance of the Materiel Used	Pregnancy Outcomes
Kivelio, 2013, [51] Switzerland, Belgium	Mid gestational model, case–control study	31 days	Mussel glue (MG) alone or combined with decellularized amnion membrane (DAM), and fibrin glue (FG) combined with decellularized amnion membrane	Large amniotic defect of 2.1 mm	Short-term outcomes, after 7 days 75% sealing membrane	80% of fetal survival for MG + DAM, 60% for MG and 40% for FG + DAM
Mogami, 2017 [52], US	Mouse model of sterile membrane rupture mechanism healing	21 days	Arg1-positive M2-macrophages. Amniotic fluid macrophages of fetal origin at the level of amnion	Small (0.47 mm) and large rupture (0.91 mm)	Mid-term evaluation at 15 days of gestation Small ruptures of the amnion closed by 24–72 h, >50% of large ruptures remained open	86% of intrauterine fetal survival rates after small rupture and 82% after a large rupture at 72 h
Lee, 2018 [53], South Korea	Micropig M-type, case–control study	114 days	Amnion-analogous medical device (AMED), a biocompatible 3D–printed device containing amniotic membrane-derived gel, compared with AmnioGraftpatch and adhesive group or decellularized human membranes (DAM) group or nonsealing group	1.2 mm	Short- and long-term evaluation AMED is easy, rapid, and is a better target to apply than an Amniopatch or DAM. Decellularized amniotic membrane gel heals wounds more than two times faster than collagen	AMED improved the preservation of the amniotic fluid, needs short surgical time for insertion, and is associated with better fetal survival and development
Engels, 2018 [54], Belgium	Case–control study	31 days	Conventional collagen (Lyostypt, B. Braun Medical N.V., Melsungen, Germany), Lyostypt soaked in fibrinogen concentrate (Haemocomplettan, CSL Behring, Breda, The Netherlands), condensed collagen from the human amniotic membrane (CCHA), Tissuepatch (Tissuemed Ltd., Leeds, UK), and Duraseal (Integra LS N.V., Zaventem, Belgium)	1.3 mm defect at 23 days of gestation	Evaluation at term CCHA and Tissuepatch had no effect on fetal survival when compared to unmanipulated control sacs (without sealant), also sealed sacs more efficiently with low fluid leakage but dissolved rapidly	Fetal survival rate is lower in the sealant groups, 72%, respectively, 78%, 77%, and 60%.
Zhao, 2022 [55], China	Mid-gestational New Zealand rabbit model, case–control study	32 days	Ultrafast photoresponsive hydrogel (1.5 s) and a 7-axis bioprinting robot to perform subaqueous in situ bioprinting in a minimally invasive approach	1.9 mm defect at 22 days of gestation	Evaluation at term 8 out of 10 patches show complete sealing. All patches were founded: 2 out of 10 patches were freely in the uterus. In 8 out of 10 cases no amniotic fluid leakage	Fetal survival rate was 72.7% in the sealing group and 81.3% in the native control group. After the rupture of membranes, no fetal weight gain
Avilla-Royo, 2023 [56], Switzerland	Swiss Alpine white Ewes model case–control	145–155 days	Elastic Mussel glue of a copolymer of poly(propylene oxide) and flexible poly(ethylene) oxide applied by an umbrella-shaped device, followed by the closure of the uterine defect	11 mm uterine and fetal defect at gestational age 56–69 days	Evaluation 10 days after introduction. Sutures and glue-induced adhesions were observed on 4 out of 8 horns. All implant sites were tightly sealed, and no fluid leakage, no amnion bands, or skin defects were observed in any of the fetuses	10 survival from 11 cases. No reported maternal or fetal complications

* First author’s name was noted.

**Table 3:** In vitro studies on human amniotic fetal membrane defect and sealing mechanisms evaluation.

Study *, Year, Country	Amniotic Membrane Tissue Model	Description of Membranes	Healing Mechanism or Material	Diameter of Membrane Defect	Possible Mechanism	Outcomes
Lee, 2020 [57], South Korea	Human amniotic membranes. Human amnion pore culture technique	39–40 weeks	Spontaneous healing mechanism	1, 2, and 3 mm	The human amnion might possibly retain pluripotent properties, such as promoting cell regeneration and natural healing membranes attributed to the amniotic epithelial stem cells (AESCs)	Cellular regrowth in the punched amniotic membrane tissue that covered the pore area within 10 days of incubation in all cases of membrane rupture by resealing small pores (<1 mm), but with no significant change in the size of the large pores (2 and 3 mm in diameter)
Kondoh, 2021 [58], Japan.	Ex vivo model of non-pregnant uterus	NS	Intracervical elastomeric sealant Hydrofit^R^ (Sanyo Chemical Industries, Ltd. Kyoto, Japan) compared with fibrin glue (0.3 mL of thrombin solution with 0.3 mL of fibrinogen solution, Bolheal (Teijin, Osaka, Japan)	NS	The sealant would have the potential to prevent the leakage of amniotic fluid in pregnancies with previable premature rupture of membranes	No amniotic fluid 15 min after application
Barrett, 2021 [59] UK	Human amniotic liquid (16–24 weeks). Human membranes	39–40 weeks	Peptide amphiphiles (PAs) conjugated with ligands for cell-adhesion (RGDS), migratory (GHK), or regenerative (GHK/RGDS) peptides assembled with amniotic fluid. PAs are represented by PAK2, PAK3, PAK4, and PAH3. PAs were applied to the surface of the membrane defect and cultured for up to 5 days with amniotic fluid replaced every 48 h	0.8 mm	PAK3 and amniotic fluid molecules form a solid membrane at the PAK3–AF interface; PAK2, PAK4, and PAH3 form a soft, liquid, or paste-like gel membrane that disintegrates after 6 h of culture	PAK3 forms a multi-layer nanofibrous network, a plug, that seals the membranes defect
Meuwese, 2022 [60], The Netherlands	Human fetal membranes 4 h–24 h after birth. A sac was formed and filled with water	NS	Crimped, froze, and crosslinked lyophilized type 1 collagen plug to obtain a highly purified collagen plug with shape memory	3 mm	Crosslinking, expanding, shape recovery, freezing, lyophilization, and crimping. The plugs triple their diameter within a minute	No further rupture of the membranes caused by the expansion of the plug The plug expanded from 1.8 mm to more than 6 mm in 60 s, more than three times its diameter.

* First author’s name was noted. NS: not specified.

## Data Availability

The data can be available to readers upon request.

## Supplementary Materials

The following supporting information can be downloaded at: https://www.mdpi.com/article/10.3390/biomedicines11071900/s1, PRISMA 2020 Main Checklist.
