# Supplementary material for: Is Spontaneous Preterm Prelabor of Membrane Rupture Irreversible? A Review of Potentially Curative Approaches
Source: Biomedicines. 2023 Jul 4;11(7):1900. doi: 10.3390/biomedicines11071900 (PMC10377155; doi:10.3390/biomedicines11071900)
Supplement: Supplementary file 1 [file biomedicines-11-01900-s001.zip › biomedicines-2430378-supplementary.pdf]

Supplementary Materials

Quality score items evaluation from animal models [1].

Item	Score Point Allocation		Score Point Allocation
Category 1: Reporting of study subject details and welfare		Category 3: Study planning quality	
1. Animal protocol approved	Reported yes = 1/no = 0	13. Study hypothesis	Reported yes = 1/no = 0
2. Species	Reported yes = 1/no = 0	14. A priori endpoint definition	Reported yes = 1/no = 0
3. Sex and age	Reported yes = 1/no = 0	15. A priori sample size calculation	Reported yes = 1/no = 0
4. Pre-study health	Reported yes = 1/no = 0	16. Reference to previous studies	Reported yes = 1/no = 0
5. Comorbidities	Reported yes = 1/no = 0	17.Inclusion/exclusion criteria	Reported yes = 1/no = 0
6. Adequate medication	Reported yes = 1/no = 0	18.Effect size/treatment effect	Reported yes = 1/no = 0
Category 2: Internal study validity		Category 4: Outcome analysis and reporting	
7. Blinding	Reported yes = 1/no = 0	19. Individual data points	Reported yes = 1/no = 0
8. Randomization	Reported yes = 1/no = 0	20. Drop outs/excluded subjects	Reported yes = 1/no = 0
9. Allocation concealment	Reported yes = 1/no = 0	21. Appropriate statistical tests	Used yes = 1/no = 0
10. Physiological parameters	Measuring reported yes = 1/no = 0	22. Potential error sources	Reported yes = 1/no = 0
11. Analysis modalities	Appropriate modalities reported [a] yes = 1/no = 0	23. Study/methodological limits	Reported yes = 1/no = 0
12. Infarct induction confirmation	Reported yes = 1/no = 0	24. Justified conclusion given	Provided yes = 1/no = 0

[a] Analysis modalities were considered appropriate when being sufficient to assess the respective research question or endpoint (see Supplementary Table S1).

Table S1. Quality evaluation for animal studies.

Article's Author Name	Quality Score Evaluation	Observations
Kivelio 2013 [2]	23	No effect size/treatment effect
Mogami 2017[3]	23	No effect size/treatment effect
Lee 2018[4]	22	No effect size/treatment effect<br>No potential error sources
Engels 2018 [5]	23	No effect size/treatment effect
Zhao 2022 [6]	22	No effect size/treatment effect<br>No potential error sources

Avilla-Royo 2022 [7]	20	No effect size/treatment effect<br>No potential error sources<br>Study /methodological limits<br>Blinding

Quality score evaluation for human and in vivo samples studies : DOWNS AND BLACK CHECKLIST modified [8]

Category 1: Selection and demographics of subjects

1. Is the hypothesis/aim/objective of the study clearly described?

2. Are the main outcomes to be measured clearly described in the Introduction or Methods section?

3. Are the characteristics of the patients included in the study clearly described ?

4. Are the interventions of interest clearly described?

5. Are the distributions of principal confounders in each group of subjects to be compared clearly described?

6. Are the main findings of the study clearly described?

Category 2: Reliability of the data

7.Does the study provide estimates of the random variability in the data for the main outcomes?

8.Have all important adverse events that may be a consequence of the intervention been reported?

9. Have the characteristics of patients lost to follow-up been described?

10. Have actual probability values been reported

Category 3 : External Validity

11. Were the subjects asked to participate in the study representative of the entire population from which they were recruited?

12. Were those subjects who were prepared to participate representative of the entire population from which they were recruited?

13. Were the staff, places, and facilities where the patients were treated, representative of the treatment the majority of patients receive?

Category 4 : Internal Validity – bias

14. Was an attempt made to blind study subjects to the intervention they have received?

15. Was an attempt made to blind those measuring the main outcomes of the intervention?

16. If any of the results of the study were based on “data dredging”, was this made clear?

17. Do the analyses adjust for different lengths of follow-up of patients, or in case-control studies,

is the time period between the intervention and outcome the same for cases and controls?

18. Were the statistical tests used to assess the main outcomes appropriate?

19. Was compliance with the intervention/s reliable?

20. Were the main outcome measures used accurate (valid and reliable)?

Category 4 : Internal Validity - confounding (selection bias)

21. Were the patients in different intervention groups (trials and cohort studies) or were the cases and controls (case-control studies) recruited from the same population?

22. Were study subjects in different intervention groups (trials and cohort studies) or were the cases and controls (case-control studies) recruited over the same period of time?

23. Were study subjects randomised to intervention groups?

24. Was the randomised intervention assignment concealed from both patients and health care staff until recruitment was complete and irrevocable?

25. Was there adequate adjustment for confounding in the analyses from which the main findings were drawn?

26. Were losses of patients to follow-up considered?

Category 5 : Power

27. Did the study have sufficient power to detect a clinically important effect ?

Article's Author Name	Quality Score Evaluation	Observations
Kwak [9]	5	Low reliability<br>Low validity<br>Reproductible<br>No external verification
Crowley [10]	5	Two randomised control trials<br>Low reliability<br>Low validity<br>No reliable<br>No external verification
Sung [11]	5	Retrospective<br>Low reliability<br>Low validity<br>Reproductible<br>No external verification
Ferianec [12]	6	Low validity<br>Reproductible<br>No external verification
Lee [13]	7	Reliable<br>Reproductible<br>Low validity
Kondoh [14]	8	Reproductible
Barrett [15]	8	Reproductible
Mewese [16]	7	Reproductible

References

1. Kringe, L.; Sena, E.S.; Motschall, E.; Bahor, Z.; Wang, Q.; Herrmann, A.M.; Mülling, C.; Meckel, S.; Boltze, J. Quality and validity of large animal experiments in stroke: A systematic review. *J. Cereb.Blood Flow Metab.* **2020** , 40(11):2152-2164. doi: 10.1177/0271678X20931062.
2. Kivelio, A.; Dekoninck, P.; Perrini, M.; Brubaker, C.E.; Messersmith, P.B.; Mazza, E.; Deprest, J.; Zimmermann, R.; Ehrbar, M.; Ochsenbein-Koelble, N. Mussel mimetic tissue adhesive for fetal membrane repair: initial in vivo investigation in rabbits. *Eur J. Obstet Gynecol Reprod Biol.* 2**013** ,171(2):240-245. doi: 10.1016/j.ejogrb.2013.09.003.
3. Mogami, H.; Hari Kishore, A.; Akgul, Y.; Word, R.A. Healing of Preterm Ruptured Fetal Membranes. *Sci Rep* .**2017** ,7(1):13139. doi: 10.1038/s41598-017-13296-1. /US
4. Lee, J.Y.; Kim, H.; Ha, D.H.; Shin, J.C.; Kim, A.; Ko, H.S.; Cho, D.W. Amnion-Analogous Medical Device for Fetal Membrane Healing: A Preclinical Long-Term Study. *Adv Healthc Mater.* **2018** ,7(18),e1800673. doi: 10.1002/adhm.201800673.
5. Engels, A.C.; Joyeux, L.; Van der Merwe, J.; Jimenez, J.; Pranpanus, S.; Barrett, D.W.; Connon, C.; Chowdhury, T.T.; David, A.L.; Deprest, J. Tissuepatch is biocompatible and seals iatrogenic membrane defects in a rabbit model. Prenat Diagn. **2018** ,38(2), 99-105. doi: 10.1002/pd.5191. Epub 2017 Dec 11. Erratum in: Prenat Diagn. 2018 May;38(6):471.

6. Zhao, W.; Hu, C.; Xu, T.; Lin, S.; Wang, Z.; Zhu, Y. Subaqueous Bioprinting: A Novel Strategy for Fetal Membrane Repair with 7-Axis Robot-Assisted Minimally Invasive Surgery. *Adv Funct Mater.* **2022**. https://doi.org/10.1002/adfm.202207496
7. Avilla-Royo, E.; Seehusen, F.; Devaud, Y.R.; Monné Rodriguez, J.M.; Strübing, N.; Weisskopf, M.; Messersmith, P.B.; Vonzun, L.; Moehrlen, U.; Ehrbar, M.; Ochsenbein-Kölble, N. In vivo Sealing of Fetoscopy-Induced Fetal Membrane Defects by Mussel Glue. *Fetal Diagn Ther*. **2022**,49(11-12),518-527. doi: 10.1159/000528473.
8. Downs, S.H.; Black, N. The feasibility of creating a checklist for the assessment of the methodological quality both of randomised and non-randomised studies of health care interventions. *J Epidemiol Community Health*. **1998**,52(6):377–384. doi: 10.1136/jech.52.6.377.
9. Kwak ,H.M.; Choi, H.J.; Cha, H.H.; Yu, H.J.; Lee, J.H.; Choi, S.J.; Oh, S.Y.; Roh, C.R.; Kim, J.H. Amniopatch treatment for spontaneous previable, preterm premature rupture of membranes associated or not with incompetent cervix. *Fetal Diagn Ther*. **2013**,33(1),47-54. doi: 10.1159/000342418.
10. Crowley, A.E.; Grivell, R.M.; Dodd, J.M. Sealing procedures for preterm prelabour rupture of membranes. *Cochrane Database Syst Rev*.**2016** ,7(7),CD010218. doi: 10.1002/14651858.CD010218.pub2.
11. Sung, J.H.; Kuk, J.Y.; Cha, H.H.; Choi, S.J.; Oh, S.Y.; Roh, C.R.; Kim, J.H. Amniopatch treatment for preterm premature rupture of membranes before 23 weeks' gestation and factors associated with its success. *Taiwan J Obstet Gynecol*. **2017**, 56(5):599-605. doi: 10.1016/j.tjog.2017.08.005.
12. Ferianec, V.; Križko, M.; Gábor, M.; Papcun, P.; Alföldi, M.; Feriancová, M. Amniopatch as an active treatment of spontaneous previable rupture of membranes. *J Matern Fetal Neonatal Med* .2**022** ,35(25):9900-9906. doi: 10.1080/1476758.2022.2072723.
13. Lee, A.Y.; Ryu, K.J.; Ahn, K.H.; Kang, D.; Geum, D.H.; Kim, B.S.; Cho, G.J.; Oh, M.J.; Kim, H.J.; Hong, S.C. Spontaneous healing of human amnion in the premature rupture of membrane model. *Placenta*. **2020** ,97,29-35. doi: 10.1016/j.placenta.2020.06.009.
14. Kondoh, E.; Kawamura, Y.; Chigusa, Y.; Mogami, H.; Ueda, A.; Hamanishi, J.; Mandai, M. Intracervical elastomeric sealant in an ex vivo model. *J Matern Fetal Neonatal Med*. 2**021**, 34(7):1109-1111. doi: 10.1080/14767058.2019.1626367
15. Barrett, D.W.; Okesola, B.O.; Costa, E.; Thrasivoulou, C.; Becker, D.L.; Mata, A.; Deprest, J.A.; David, A.L.; Chowdhury, T.T. Potential sealing and repair of human FM defects after trauma with peptide amphiphiles and Cx43 antisense. *Prenat Diagn.* **2021** ,41(1),89-99. doi: 10.1002/pd.5826.
16. Meuwese, R.T.C.; Versteeg, E.M.M.; van Drongelen ,J.; de Hoog, D.; Bouwhuis, D.; Vandenbussche, F.P.H.A.; van Kuppevelt, T.H.; Daamen, W.F. A collagen plug with shape memory to seal iatrogenic fetal membrane defects after fetoscopic surgery. *Bioact Mater*. **2022** , 20,463-471. doi: 10.1016/j.bioactmat.2022.06.007.
